# A computational approach for investigating Coulomb interaction using Wigner–Poisson coupling

**DOI:** 10.1007/s10825-020-01643-x

**Published:** 2021-01-21

**Authors:** Majid Benam, Mauro Ballicchia, Josef Weinbub, Siegfried Selberherr, Mihail Nedjalkov

**Affiliations:** 1 Institute for Microelectronics, TU Wien, Vienna, Austria; 2 Christian Doppler Laboratory for High Performance TCAD, Institute for Microelectronics, TU Wien, Vienna, Austria

**Keywords:** Coulomb interaction, Wigner formalism, Entanglement, Purity

## Abstract

Entangled quantum particles, in which operating on one particle instantaneously influences the state of the entangled particle, are attractive options for carrying quantum information at the nanoscale. However, fully-describing entanglement in traditional time-dependent quantum transport simulation approaches requires significant computational effort, bordering on being prohibitive. Considering electrons, one approach to analyzing their entanglement is through modeling the Coulomb interaction via the Wigner formalism. In this work, we reduce the computational complexity of the time evolution of two interacting electrons by resorting to reasonable approximations. In particular, we replace the Wigner potential of the electron–electron interaction by a local electrostatic field, which is introduced through the spectral decomposition of the potential. It is demonstrated that for some particular configurations of an electron–electron system, the introduced approximations are feasible. Purity, identified as the maximal coherence for a quantum state, is also analyzed and its corresponding analysis demonstrates that the entanglement due to the Coulomb interaction is well accounted for by the introduced local approximation.

## Introduction

Collective phenomena such as Coulomb interaction play a dominant role in determining the behaviour of classical microelectronic devices [1]. Conventionally, this requires coupling the Boltzmann equation (or the macroscopic models derived from it, such as the drift-diffusion equations) with the Poisson equation. Sophisticated particle models have been developed, where the mesh-dependent Boltzmann–Poisson model is accomplished by short range interactions [2] to correctly represent the Coulomb interaction without using computationally expensive methods such as molecular dynamics [3]. Cutting-edge nanoelectronic devices are usually described in terms of single-electron quantum mechanics, or in terms of Schrödinger–Poisson models [4], up to the exhaustive many-body Schrödinger equation [5] used in quantum chemistry and materials science.

Usually, nonequilibrium electron dynamics is sufficient to describe the charge transport in general nanometer structures, governed by boundary conditions. However, recent efforts to develop novel devices by using alternative operating principles focus on coherence and entanglement [6]. A classical and fundamental case for the development of quantum computing approaches is the evolution of two electrons in two adjacent quantum wires [7]. The initial state can be represented by two separate electrons, or can be correlated, if the electrons are indistinguishable [8, 9]. However, the core effect of the electron–electron interaction, occurring during the common evolution in the adjacent wires, is the process of entanglement [10, 11], to the degree that such structures are collectively known as so-called Coulomb entanglers. Entanglement is measured by different quantities [12], such as the von-Neumann entropy $tr(\rho ln(\rho ))$ [13] or the linear entropy $tr(\rho (1-\rho ))$ [14], where $\rho$ represents the density matrix. Similarly, one can use the deviation of $tr(\rho ^2)$ from unity, which serves as a convenient heuristic measure of the purity of a state, based on the fact that for pure states $\rho =\rho ^2$ holds.

Coherence is an underlying quantum concept. The quantification theory of coherence has recently been developed based on the quantification theory of entanglement [15, 16]. While the two concepts describe very different physical notions, the two theories have a common mathematical foundation. They have been developed in the framework of operator mechanics, in terms of Hilbert spaces, eigenbasis sets, and tensor products. Accordingly, simulating quantum systems relies on computational approaches of wave mechanics. In particular, the time-dependent Schrödinger equation with exact Coulomb interaction is solved [17], or approximated by resorting to coupling schemes to the Poisson equation [18].

The Wigner formalism offers an alternative description of quantum mechanics in terms of phase space. The formalism is widely applied [19, 20], and offers features such as an intuitive and heuristic picture of pure quantum processes by providing a seamless transition to classical evolution. It has been shown recently that the formalism provides a legitimate theoretical framework which presents the basic notions of the quantification theory of coherence in phase space terms [21]. Coherent single-electron dynamics as well as effects of decoherence can be analyzed with the help of a stochastic computational approach [22]. The applied quantum Monte Carlo approach samples the single-electron evolution in terms of particles equipped with a sign, which are generated by the Wigner potential and can be annihilated in a common phase-space cell [23]. The corresponding algorithm is independent of the dimensionality of the computational problem. The phase space $$({\mathbf{r}},{\mathbf{k}})$$ can correspond to the coordinates $$(x,k_x$$, $$y,k_y$$, $$z,k_z)$$ of a single electron in three dimensions, or to three one-dimensional (1D) electrons $$(x_1,k_{x1})$$, $$(x_2,k_{x2})$$, and $$(x_3,k_{x3})$$, or denote the set $${\mathbf{r}}_1,{\mathbf{r}}_2,{\mathbf{k}}_1,{\mathbf{k}}_2$$ of two 2D or 3D electrons.

As entanglement considers at least two quantum objects, the dimensionality of the phase space increases, and the Coulomb interaction between the evolving electrons must be accounted for. However, the numerical efforts related to the computation of the Wigner function $f_w({\mathbf{r}},{\mathbf{k}},t)$ and the most dimensionally-dependent object in the theory, the Wigner potential $V_w({\mathbf{r}},{\mathbf{k}},{\mathbf{k}}',t)$, rapidly rise with the increase of the dimensionality. Furthermore, in the considered case of a Coulomb entangler [10], the Wigner potential, which in this two-electron case depends on $2\times 6$ $({\mathbf{r}},{\mathbf{k}},{\mathbf{k}}')$ arguments for 2D structures, must be updated during the evolution in timesteps of the order of one femtosecond. This gives rise to an enormous computational effort and motivates the development of approximative methods.

Separating the dynamically evolving Coulomb interaction from the stationary potential and representing the former in terms of an electric force will indeed reduce the computational burden; the Wigner equation states that a force-less Liouville operator, acting on the Wigner function, equals the integral on ${\mathbf{k}}'$ between the Wigner potential and the Wigner function [24]. The integral possesses the nice property of turning into the force term of the Liouville operator for electric potentials with up to quadratic spatial dependence [25]. Therefore, if the electric field varies slowly in the spatial region in the vicinity of an electron wavepacket, its evolution is accelerated by the local electric force, as in the Boltzmann case. This property has motivated Gehring and Kosina to introduce a low-pass filter [26] in order to separate the slowly varying component of the electric field and complement the Liouville operator with a force term. This reduces the effect of the Wigner potential, because a part of it is represented by a force. The accelerated Newtonian trajectories can be efficiently computed, which significantly reduces the computational demands. Thus, a feasible approach for the analysis of entanglement is offered by the spectral decomposition of the Coulomb interaction, which is effectively represented by the electric force.

The Poisson equation has been incorporated in the analysis of the Wigner self-consistent potential since the early applications of the Wigner equation in device modeling [27, 28], thus there is a well-established approach to the self-consistent evolution. While in classical device modeling, the transport equation provides the number of electrons, which in turn updates the electric field used in the next timestep of the evolution, in the quantum case, this scheme contains an implicit assumption related to the fact that electrons are not point-like particles anymore, and indeed spread over at least several nanometers. The quantum electron is non-local with respect to the spatial mesh required for the solution of the Poisson equation and, therefore, the spread of the density of a single electron should cause negligible perturbation on the density of adjacent mesh nodes, which are used to calculate the local force. This is true if the number of electrons is very large, which is an important assumption in the case of modern devices. However, when considering the case of two distinguishable interacting electrons, the density distribution of the first electron provides the electric field for the second electron, and vice versa. Accordingly, the density of each electron must be ruled out in calculating its effective electric field in order to avoid self-action. The computational problem of several interacting electrons is thus entirely different from the commonly used many-body counterpart.

In this work, we present a computationally efficient approach for a Wigner–Poisson coupled scheme in order to investigate the evolution of interacting electrons. For this purpose, it is important to inspect conditions which allow adjusting the non-locally acting Wigner potential (the electron–electron Coulomb interaction) to a local action of the electric force.

In Sect. 2, we formulate the computational problem by describing the physical system, the evolutionary process, and the computational aspects. The effect of the spectral decomposition of the potential on the mean values of the physical quantities together with an evaluation of the error is presented in Sect. 3. The non-locality of the action is another underlying quantum concept, and thus, the impact of the local field approximation on the capability of the approach to simulate entanglement is analyzed through simulations addressed in Sect. 4.

## Computational problem

**Fig. 1:**
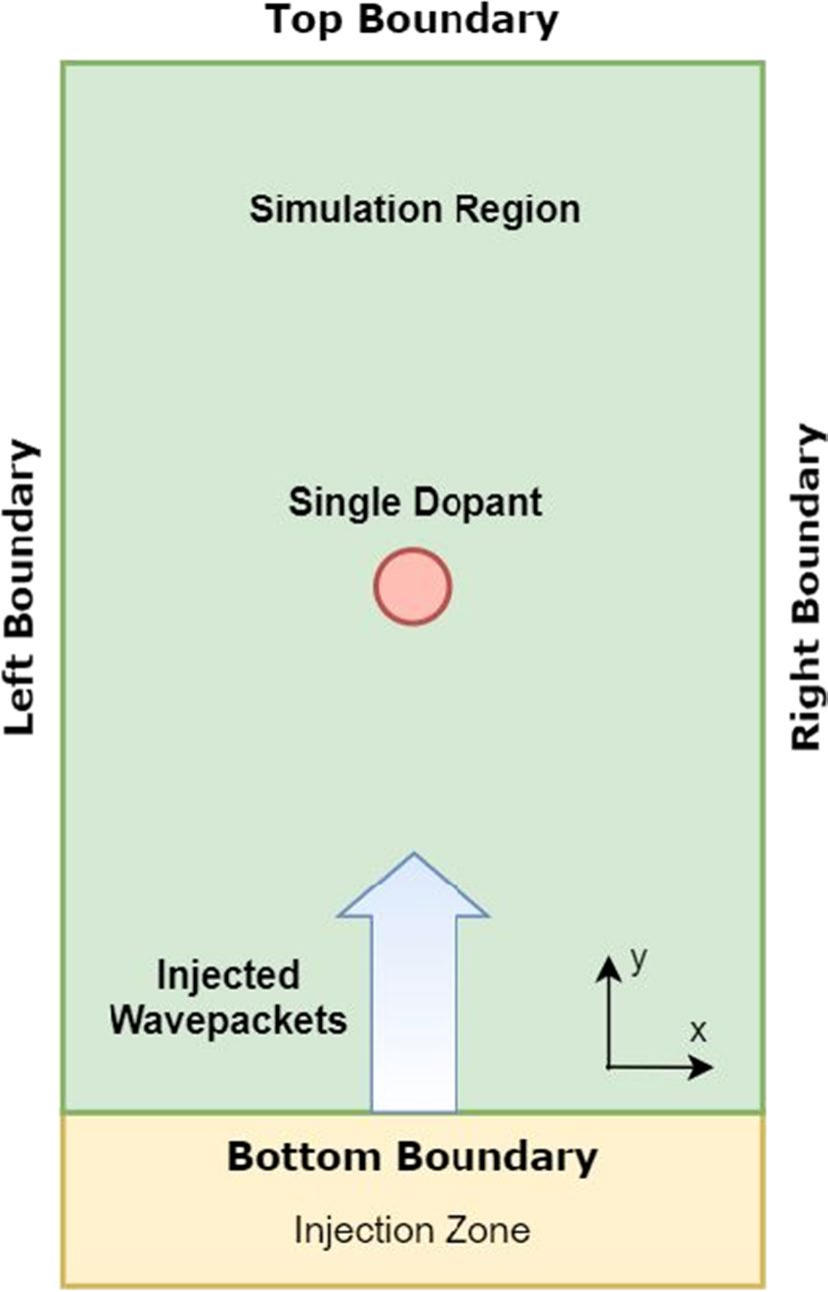
The simulation and injection setup of the 2D nanowire

In the Wigner Ensemble Monte Carlo (WEMC) simulation scheme, the injection of electrons is implemented through the utilization of minimum uncertainty wavepackets [21]. The Wigner function, representing the initial condition, is calculated from a physically valid wavefunction. The Gaussian minimum uncertainty wavepacket is defined by

1 $$\begin{aligned} \psi _G({\mathbf{x}}, {\mathbf{k}},t_0)= N e^{-({\mathbf{r}}-{\mathbf{r}_0})^2\sigma ^{-2}} e^{-(\mathbf{q}\Delta k - {\mathbf{k}_0})^2 \sigma ^2}, \end{aligned}$$

where *N* represents a normalization constant, and $r_0$, $k_0$ and $\sigma$ represent the mean position, the mean wavevector, and the standard spatial deviation, respectively. Each injected wavepacket consists of many numerical particles and represents a single electron. As shown in Fig. 1, the injected wavepackets are first generated in the so-called injection zone, where the initial distributions of position and momentum for the numerical particles in each wavepacket follow Gaussian distributions. In this regard, we can either inject wavepackets separately, i.e., without any Coulomb interaction considered, or inject pairs of coupled wavepackets, i.e., taking Coulomb interaction into account. In order to obtain a steady-state picture for physical quantities of interest, the coupled pairs are injected independently of other couples in intervals of $t_{inter}$ (the time between the injection of one pair and the next pair). Furthermore, the time between the injection of two particles in the same pair is denoted by $t_{intra}$, and is of high importance in the description of the coupled-injection experiments presented here, as will be discussed later.

A Coulomb entangler is a synthesized system of two electrons, represented by two equivalent minimum uncertainty wavepackets (i.e. two equivalent electrons), injected into a nanowire in the absence of dopants in the simulation region. The external potential is ignored, which ensures a valid analysis of the entanglement as the influence of the environment is eliminated. The quantum character of the evolution is thus specified only by the initial conditions, which obey the uncertainty relations.

The formal description of the evolution of a single electron requires a 4D phase space for a 2D simulation region. A coupled electron–electron pair, in terms of the 2D Wigner equation, involves a force-less Liouville operator acting on the Wigner function $f_w$ in the 8D phase space of position ${\mathbf{r}}={\mathbf{r}}_1, {\mathbf{r}}_2$ and wavevector ${\mathbf{k}}={\mathbf{k}}_1, {\mathbf{k}}_2$, taking the coordinates of both electrons into account. According to the signed-particle method [22], the mean value of a physical quantity is determined by a stochastic sampling by Monte Carlo techniques, where samples represent numerical particles. A numerical particle, in the here considered coupled pair, has eight coordinates and follows a Newtonian trajectory $({\mathbf{r}}(t), {\mathbf{k}}(t)={\mathbf{k}})$, which is parameterized by the time line in the phase space and uniquely determined by its initial point $({\mathbf{r}},{\mathbf{k}})$. The evolving trajectory does not *feel* the action of the Wigner potential $V_w({\mathbf{r}},{\mathbf{k}},{\mathbf{k}}')$, which determines the rules for generation of novel signed particles. In other words, a particle in $({\mathbf{r}},{\mathbf{k}})$ is associated with a probability to generate novel particles in $({\mathbf{r}},{\mathbf{k}}')$. The sign of novel particles and their initial point depends on the Wigner potential at $({\mathbf{r}},{\mathbf{k}})$, as well as on the sign of the parent particle. Apart from the fact that a numerical particle has eight coordinates, the algorithm is the same as for the evolution of a single electron, represented by numerical particles in a 4D phase space. However, the computational burden dramatically increases for the 8D case, which is well-illustrated by the fact that the first stochastic methods for 1D structures (2D phase space), such as resonant-tunneling diodes, were developed two decades ago [29], and it took around fifteen years before the first 4D phase space simulations were conducted [30].

Regarding entanglement, one approach is to calculate the reduced Wigner function for the first electron $f_{r1}$, which is obtained by integrating $f_w$ on the coordinates of the second electron $({\mathbf{r}}_2,{\mathbf{k}}_2)$. Furthermore, if the two electrons were non-interacting, it holds that the 8D Wigner function is given by the product of the 4D Wigner functions of two electrons $f_w=f_{w1} \times f_{w2}$, in which $f_{w1}=f_{r1}$, $f_{w2}=f_{r2}$, and this property remains during the time evolution. Thus, it is sufficient to simulate the electron of interest, a 4D problem, without the need to consider the second electron.

The Wigner potential $V_{w,e-e}$ for the electron–electron interaction is thus a 12D quantity (see Sect. 1) which depends on the coordinates of the two electrons. However, a fixed Coulomb potential, such as one of a dopant, provides $V_{w,c}$, a 6D quantity, depending on the coordinates of a single electron. In other words, while $V_{w,e-e}$ acts on $f_w$ by involving the coordinates of the two electrons, a fixed Coulomb potential appears as $V_{w,c1}+V_{w,c2}$, in which the first (second) term involves only the first (second) electron coordinates of $f_w$. It follows that, in the absence of electron–electron interaction, the 12D Wigner equation decouples into two independent 6D equations.

The above considerations encourage the local field approximation in which the term $V_{w,e-e}$ is approximated by the corresponding forces $\mathbf{F}_1$ and $\mathbf{F}_2$, acting on the first and the second electron, respectively. From a formal point of view, a decoupling is still not possible, as $\mathbf{F}_1$ depends on the position variables of the second electron, and vice versa. However, as in the case of classical transport modeling, one can assume constant values for the forces in timesteps of $\Delta t=1\mathrm \,fs$ and update $\mathbf{F}_1$ and $\mathbf{F}_2$ at the end of each timestep. During a timestep, $\mathbf{F}_1$ ($\mathbf{F}_2$) depends only on the coordinates of the first (second) electron and the description of each of them becomes 4D. The description remains 4D also in the case of an external potential as they interact with any of the electrons independently of the other electrons, which greatly reduces the computational burden.

In this way the two-electron Wigner equation is reduced to two coupled equations solved independently during consecutive time steps. The update of the forces at the end of each time step establishes the coupling due to the Poisson equation, which introduces information about the solution of the first of the coupled equations into the second coupled equation and vice versa. Even if an initially decoupled system is considered, corresponding to an initial condition represented by a product of two single-electron Wigner functions, the interaction entangles the two electrons. The two-electron Wigner function evolved in time is still a product of the solutions of the two equations, but each of these solutions is now a function which depends on both sets of coordinates labeled by 1 or 2, respectively, established via the consecutive update of the forces. That is, the two-electron Wigner function cannot be decomposed into a product of two functions $f_1\times f_2$ depending solely on the coordinates 1 or 2, respectively. The system is not separable anymore so that a single-electron distribution is obtained only after integration over the other set of coordinates.

## Wigner potential decomposition

The local field approximation is essential for the developed Wigner–Poisson coupling scheme. In this section, we provide an analysis of the effect of the potential decomposition and show that the devised electron–electron local field approximation is feasible, if the electrons are initially at a *moderate* distance and do not *overlap significantly* (also during the evolution), see Sect. 4.

The Wigner function [31], as a quasi-probability measure in phase space, is obtained by the Fourier transform of the density matrix expressed in the mean and difference of coordinates:

2 $$\begin{aligned} f_w({\mathbf{r}},{\mathbf{k}},t) = \frac{1}{(2\pi )^2}\int _{-\infty }^{+\infty }d\mathbf{s}e^{-i{\mathbf{k}}.\mathbf{s}}\rho \big ( {\mathbf{r}}+\frac{\mathbf{s}}{2},{\mathbf{r}}-\frac{\mathbf{s}}{2} ,t) \end{aligned}.$$

For a finite region of interest, the position and momentum vectors can be discretized as ${\mathbf{r}} \equiv (x \Delta x, y \Delta y)$ and ${\mathbf{k}} = \mathbf{q} \Delta k \equiv (p \frac{\pi }{M \Delta x}, q \frac{\pi }{N \Delta y})$, respectively, with the physical region of interest represented by $\mathbf{L} \equiv (M \Delta x, N \Delta y)$. Without any loss of generality, mesh spacings in both directions are assumed to be identical for the remainder of this work, i.e., $\Delta x = \Delta y$. Furthermore, a function of phase space is represented as *f*(*x*, *y*, *p*, *q*), removing the constant parts to improve readability.

The Wigner equation, in the discrete form, will then follow as:

3 $$\begin{aligned}&\left( \frac{\partial }{\partial t}+ \frac{\hbar \mathbf{q}\Delta k}{m^*}\nabla _{{\mathbf{r}}} \right) f_w({\mathbf{r}},\mathbf{q},t)\nonumber \\&\quad = \sum _{\mathbf{q}} V_w({\mathbf{r}},\mathbf{q}-\mathbf{q'}) f_w({\mathbf{r}},\mathbf{q'},t) \end{aligned}.$$

The fully discretized Wigner potential, which is of central importance in the signed-particle method, will then become:

4 $$\begin{aligned}&V_w(x,y,p,q) = \frac{1}{i\hbar M N} \sum _{m= - \frac{M}{2}}^{\frac{M}{2}-1}\sum _{n=- \frac{N}{2}}^{\frac{N}{2}-1}e^{-i(pm\frac{\pi }{M}+qn\frac{\pi }{N})}\nonumber \\&\quad \times \bigg [ V \big ( x + \frac{m}{2} , y + \frac{n}{2} \big ) - V \big ( x - \frac{m}{2} , y - \frac{n}{2} \big ) \bigg ] \end{aligned}.$$

Using the properties of the 2D discrete Fourier transform, and assuming the potential to be periodic, it is proved that the Wigner potential can be given by:

5 $$\begin{aligned} V_w(x,y,p,q)&=  \frac{1}{i\hbar M N} \bigg \{ e^{i2(\frac{\pi xp}{M}+\frac{\pi yq}{N})} \hat{V}(2p,2q)\nonumber \\& \quad - \big [ e^{i2(\frac{\pi xp}{M}+\frac{\pi yq}{N})} \hat{V}(2p,2q) \big ]^* \bigg \} \end{aligned}.$$

Using Euler’s formula, Eq. 5 can be rewritten in polar form as:

6 $$\begin{aligned} V_w(x,y,p,q)&=  \frac{2}{\hbar M N} A(2p, 2q)\nonumber \\&\quad \times sin \big [ \phi (2p,2q) +2\frac{\pi xp}{M} + 2\frac{\pi yq}{N} \big ] \end{aligned}.$$

Applying the decomposition, the potential operator on the right-hand side (RHS) of Eq. 3 can be represented as:

7 $$\begin{aligned} Qf_w(x,y,p,q)&= \sum _{p',q'} V_w(x,y,p',q') f_w(x,y,p-p',q-q')\nonumber \\&=\sum \limits _{|q'|,|p'|\le \frac{q_c}{2}} + \sum \limits _{|q'|,|p'|> \frac{q_c}{2}} \nonumber \\&= Q_{cl}f_w+ Q_{qm}f_w\end{aligned}$$

$Q_{cl}$ and $Q_{qm}$ are the classical and quantum mechanical parts of the potential operator, respectively. As the mesh spacing is identical in both directions, we can specify a common cut-off wavenumber $q_c$ , which corresponds to a more practical parameter, namely the cut-off wavelength, through $\lambda _{c} = \frac{2\pi }{q_{c} \Delta k}$.

Cut-off parameters determine the sharpness of the corresponding low-pass filter, which is the heart of the decomposition scheme. The higher the cut-off wavenumber, the lower the cut-off wavelength, and thus the sharper the low-pass filter. In other words, lower values of $\lambda _c$ result in near-classical functioning of the system, while higher values of $\lambda _c$ demonstrate the near-quantum behavior.

Using Lagrange’s mean value theorem, the classical component of the potential can be calculated, after some manipulation, as:

8 $$\begin{aligned} V_{cl}(x,y)&= \frac{1}{ M N} \sum \limits _{|q|,|p|\le q_c} \hat{V} (p,q) e^{i(\frac{\pi xp}{M} +\frac{\pi yq}{N})}\nonumber \\&= \sum _{m= - \frac{M}{2}}^{\frac{M}{2}-1}\sum _{n=- \frac{N}{2}}^{\frac{N}{2}-1} V(m,n) \nonumber \\&\quad \times \Bigg ( \frac{\sin [\frac{\pi q_c(x-m)}{M}]}{\pi (x-m)}. \frac{\sin [\frac{\pi q_c(y-n)}{N}]}{\pi (y-n)} \Bigg ) \end{aligned}.$$

In order to calculate $$V_{cl}$$, a convolution of real functions, the potential *V*(*x*, *y*) and the *sinc* functions acting as low-pass filters, must be evaluated. As shown in Eq. 8, $$V_{cl}(x,y)$$ is the slowly varying part of the potential calculated by filtering out the high frequency components. In contrast, $$V_{qm}(x,y)$$ contains only the high-frequency components and represents the rapidly varying part of *V*(*x*, *y*). Being aware of $$ V(x,y) = V_{cl}(x,y) + V_{qm}(x,y)$$, it is straightforward to show that:

9 $$\begin{aligned}V_{qm,w}(x,y,p,q) &= \frac{1}{i\hbar M N}  \sum _{m= -\frac{M}{2}}^{\frac{M}{2}-1}\sum _{n=-\frac{N}{2}}^{\frac{N}{2}-1} e^{-i(\frac{\pi mp}{M}+\frac{\pi qn}{N})} \nonumber \\&\quad \times \left [ V \left ( x + \frac{m}{2} , y + \frac{n}{2} \right ) - V ( x - \frac{m}{2} , y - \frac{n}{2} ) \right ] \end{aligned}$$

Therefore, through the introduction of a low-pass filter, the potential profile is decomposed into a slowly varying classical component and a rapidly varying quantum mechanical component, and the Wigner equation is modified as follows [32]:

10 $$\begin{aligned}&\left( \frac{\partial }{\partial t}+ \frac{\hbar \mathbf{q}\Delta k}{m^*}\Delta _{{\mathbf{r}}} - \frac{1}{\hbar } \Big [\Delta _{{\mathbf{r}}} V_{cl}({\mathbf{r}})\Big ] \Delta _{\mathbf{q}} \right) f_w({\mathbf{r}},\mathbf{q},t)\nonumber \\&\quad = \sum _{\mathbf{q}} V_{qm,w}({\mathbf{r}},\mathbf{q}-\mathbf{q'}) f_w({\mathbf{r}},\mathbf{q'},t) \end{aligned}.$$

As shown on the left-hand side (LHS) of the modified Wigner equation (Eq. 10), the classical component of the potential gives rise to a local force term, which is calculated using the finite difference method. Furthermore, the new Wigner potential ($V_{qm,w}$) on the RHS of the equation is calculated from the non-local quantum mechanical component of the potential ($V_{qm}$).

**Fig. 2:**
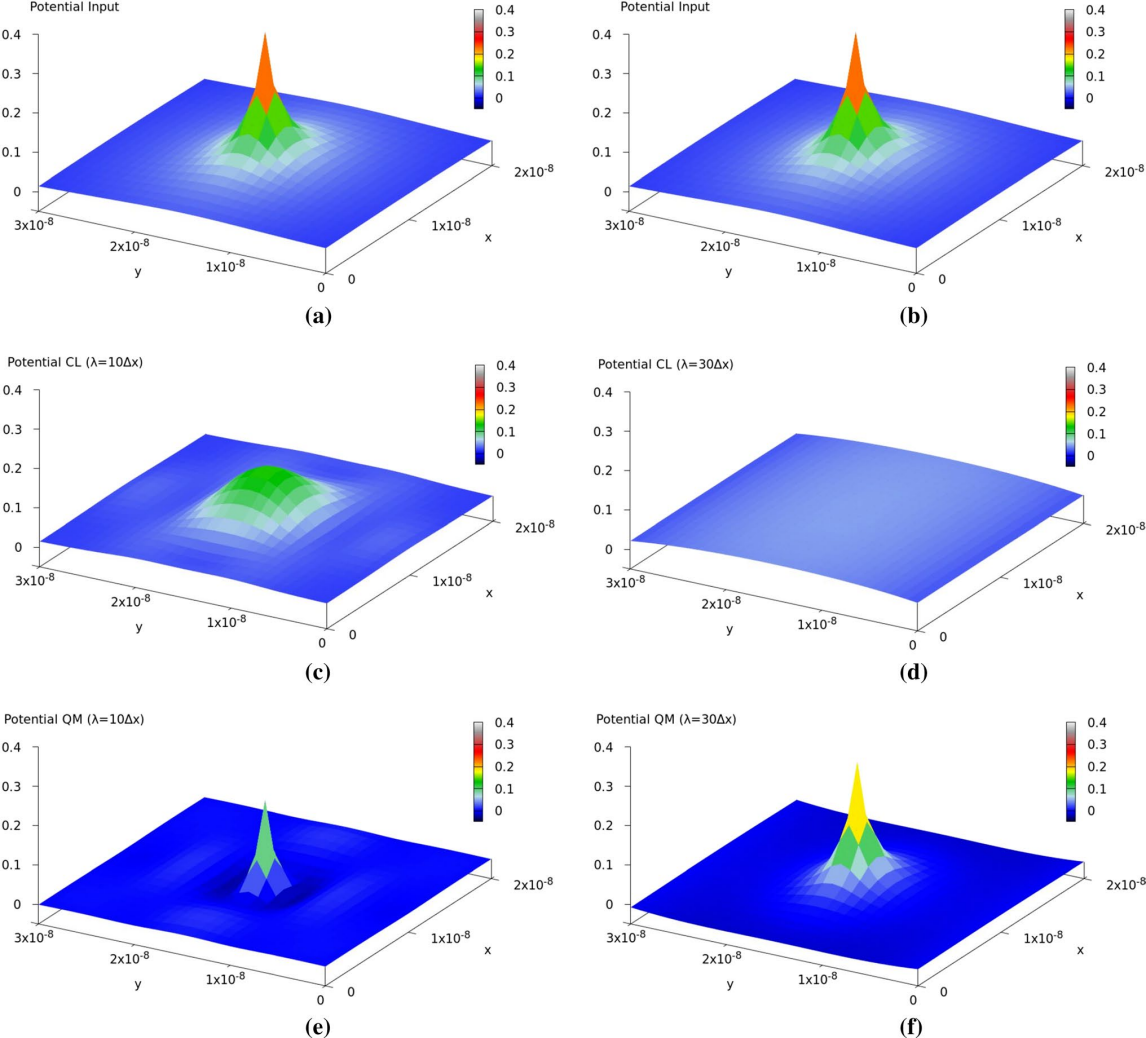
The potential input (**a**, **b**) and its classical (CL) (**c**, **d**) and quantum mechanical (QM) (**e**, **f**) components for a single charge in the center of a $20\,nm \times 30\,nm$ region for $\lambda _c = 10\Delta x$ (left figures), and $\lambda _c = 30\Delta x$ (right figures). Units are in *eV*

The results of such a potential decomposition are illustrated in Fig. 2 for $\lambda _c=10\Delta x$ (close to classical case) and $\lambda _c=30\Delta x$ (close to quantum case), respectively.

Based on this potential setup of the considered simulation domain (see Fig. 1), electrons are being injected from the bottom boundary: The simulation region does not contain any dopants (in contrast to the simulations discussed in Sect. 3), but Gaussian minimum uncertainty wavepackets (representing electrons) are injected into the region (in the $+y$ direction).

**Fig. 3:**
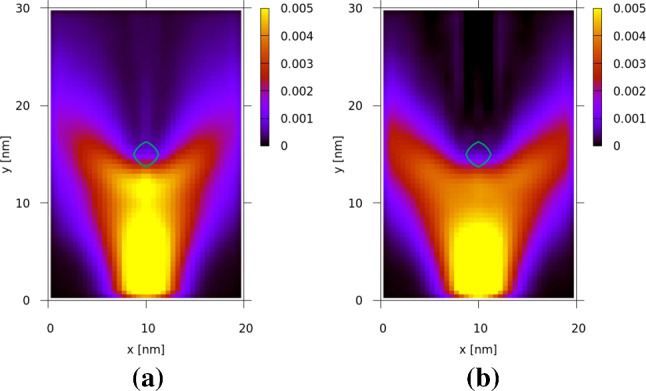
The particle density (a.u.) for **a** $\frac{\lambda _c}{\Delta x}\mathrm \,=\mathrm \,10$, and **b** $\frac{\lambda _c}{\Delta x} = 30$

All boundary conditions are treated as *absorbing*, so that the particles are not exposed to boundary potentials. The results for the density, as shown in Fig. 3 for $\lambda _c =10\Delta x$ and $\lambda _c=30\Delta x$ at $t=95\mathrm \,fs$, demonstrate the interaction of the particles with the single dopant in the center.

**Fig. 4:**
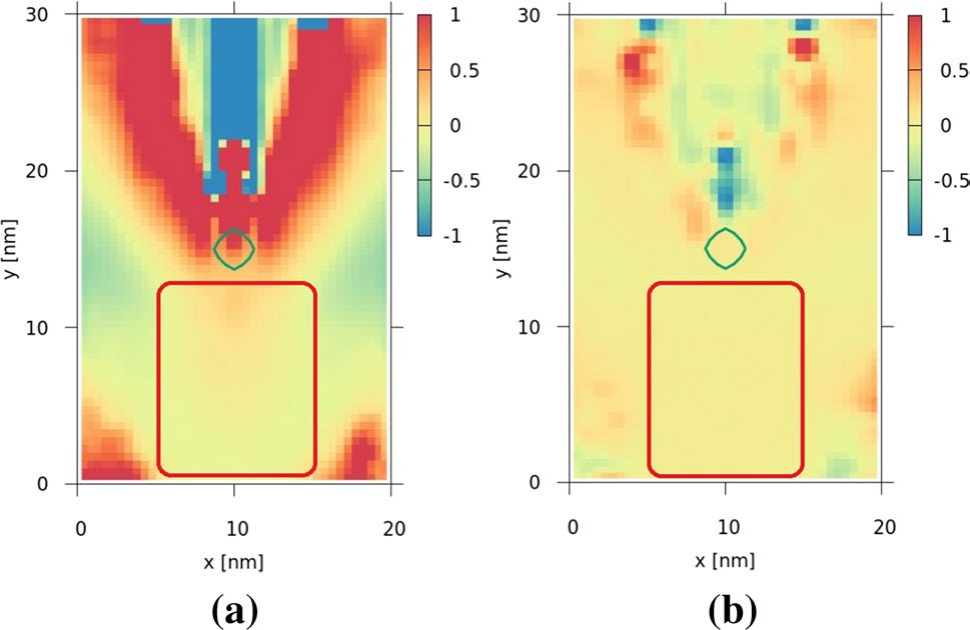
The error ratio of particle density for **a** $\frac{\lambda _c}{\Delta x} = 10$, and **b** $\frac{\lambda _c}{\Delta x} = 30$

For the above-mentioned values of the cut-off wavelength, the density values at each point in the physical region are compared in Fig. 4 to the corresponding density values of the pure quantum case and an error ratio is considered as follows [32]:

11 $$\begin{aligned} Err_{D} (x_i, y_j) = \frac{D_{\lambda _c}(x_i, y_j) - D_{qm}(x_i, y_j)}{D_{qm}(x_i, y_j)} \end{aligned}.$$

$D_{qm}$ represents the density values at each point in the physical mesh in the case of no potential decomposition, where the potential is considered to have only a quantum mechanical component and no classical component. $D_{\lambda _{c}}$, on the other hand, refers to the case where a corresponding value of $\lambda _{c}$ is chosen for the potential decomposition. The average value of $Err_{D} (x_i, y_j)$ in a time interval from $t=0\mathrm \,fs$ to $t=250\mathrm \,fs$, illustrated in Fig. 3, lies fairly close to zero ($Err_{D}\le 0.2$) for almost all the points in the region designated by the red area $x\in (5\mathrm \,nm,15\mathrm \,nm)$, $y\in (0\mathrm \,nm,12\mathrm \,nm)$. The decrease in the error is also noticeable as we increase the cut-off wavelength.

Even for the near classical case, when the electrostatic force replaces the Wigner potential, the error values remain small in the designated region. Therefore, if the second electron is in this region, the electrostatic force can be used as a decent approximation to replace the Coulomb potential of the coupled electron.

In summary, the parent particles are exposed to an electrostatic force arising from the action of the Wigner potential. Therefore, the updated coordinates of the numerical particles in the phase space result in updated rules for generating novel numerical particles, as well as for the local force in each timestep. Using the Monte Carlo approach described in [32], the momentum values are more efficiently updated on the momentum grid, but no longer have a constant value throughout the simulation.

## Coulomb entangler

In this section, we compute the evolution of the Wigner function equivalence of $Tr(\rho ^2)$, called purity, for the two reduced Wigner functions, corresponding to the two electrons, to study the impact of the Coulomb interaction.

The 2D simulation setup for this purpose consists of a quantum wire with dimensions $30\mathrm \,nm \times 60 \mathrm \,nm$. Without loss of generality, the dimensions of the simulation domain have been increased in comparison to the experiments in Sect. 3 in order to have sufficient spacing between different electron injections. All physical quantities of interest remain constant in the *z*-direction, and the wire has no dopants or other charges at the start of the experiment. The electrons are injected from the bottom boundary of the nanowire and are directed towards $+y$.

The analysis of the effect of the Coulomb interaction on the purity of the two electron states was carried out by injecting two minimum uncertainty wavepackets with a $t_{intra}$ (see Sect. 2) of $70 \,\mathrm fs$. The two electrons have the same mean velocity, i.e., about $0.25 \,\mathrm nm/s$, corresponding to $k_{0,y} = 0.42 \mathrm \,nm^{-1}$, and a positional uncertainty $\sigma _x =\sigma _y = \sigma = 3 \,\mathrm nm$. During the injection, the spatial spreading, inherent in the two Gaussian distributions of both momentum and position states, is suppressed. Therefore, the wavepackets evolve as *hard spheres* with a constant velocity equal to their mean velocity until time $t=140 \, \mathrm fs$, when both of them are fully inside the quantum wire (simulation region) at a distance of $35 \, \mathrm nm$ between their centers. At $t=140 \, \mathrm fs$ the spatial spreading is switched on and quantum evolution starts for both wavepackets. This scenario complies with the conditions for the local field approximation, discussed in Sect. 3. Firstly, the centers of the wavepackets maintain the distance (i.e. $35 \, \mathrm nm$), so that they can overlap only due to natural spreading. The distance ($35 \, \mathrm nm$) between the wavepackets is chosen to be in the order of the *y*-extent of the simulation domain ($30 \, \mathrm nm$), which is consistent with the low error region indicated by the red rectangle in Fig. 4. Moreover, the electron injection process enables insights into the sensitivity of the purity to unphysical effects. For this purpose, the portion of any electron that has already been injected into the simulation domain is normalized to unity. This portion of the electron is a legitimate classical state as it is non-negative, however, it is not eligible from a quantum-mechanical point of view, as it violates the uncertainty relation.

**Fig. 5:**
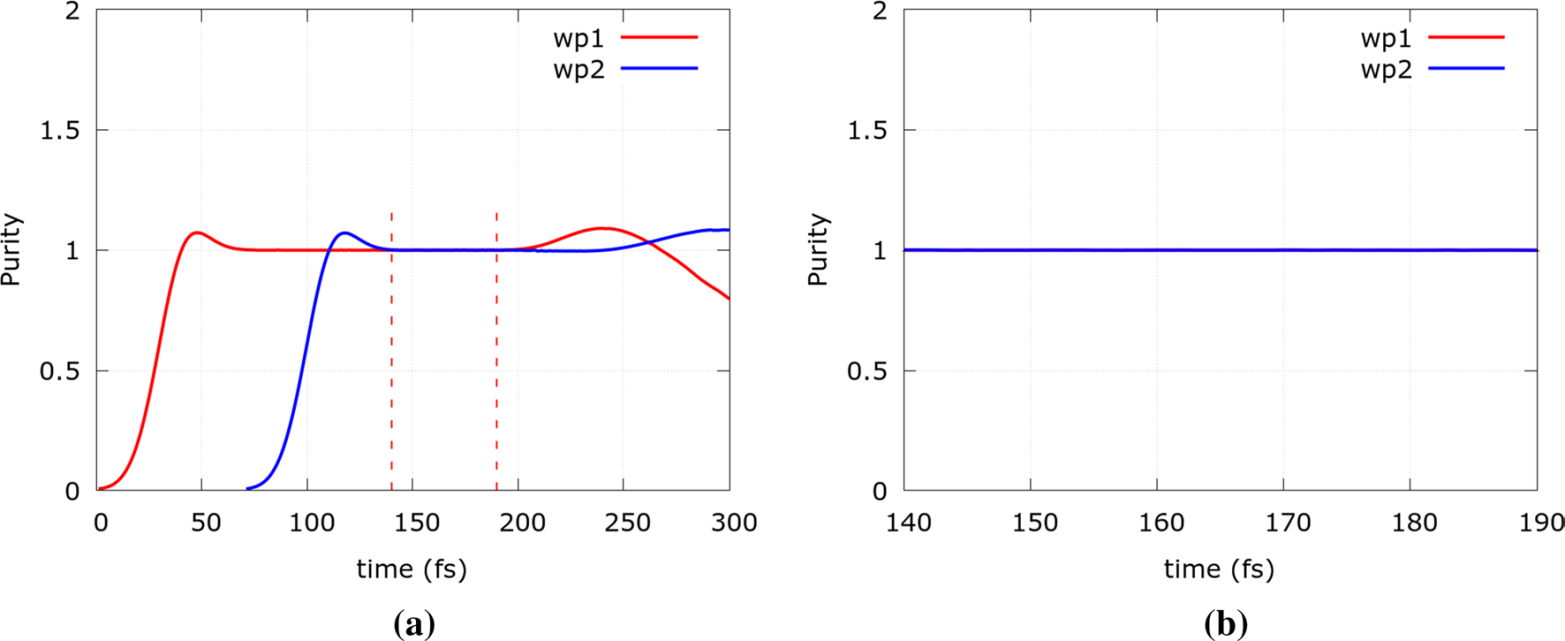
Time evolution of the purity of the non-interacting wavepackets (WP, i.e., electron) for **a** the entire evolution time (dashed red lines indicate time interval shown in **b**), and **b** the time interval from $t=140 \mathrm \,fs$ to $t=190 \mathrm \,fs$ when both electrons are inside the quantum wire (Color figure online)

Two simulation experiments were carried out. In the first one only the spatial spreading of the wavepackets is activated to simulate the quantum evolution of the two independent wavepackets, while in the second one at $t=140 \, \mathrm fs$ the Coulomb repulsion is also activated. In both cases, the purity, i.e.,

12 $$\begin{aligned} Tr(\rho ^2)_{1}(t)&=  \int d{\mathbf{k}}_1 d{\mathbf{r}}_1 f_{w1}({\mathbf{r}},{\mathbf{k}},t)\nonumber \\&=  \int d{\mathbf{k}}_1 d{\mathbf{r}}_1 \left( \int d{\mathbf{k}}_2 d{\mathbf{r}}_2f({\mathbf{r}},{\mathbf{k}},t)\right) \nonumber \\&=  Tr(\rho ^2)_{2}(t) \end{aligned}$$

is evaluated. Here we recall the definition of the two-electron Wigner function (Eq. 2) and that ${\mathbf{r}}={\mathbf{r}}_1,{\mathbf{r}}_2$ and ${\mathbf{k}}={\mathbf{k}}_1,{\mathbf{k}}_2$. Taking the trace of $\rho ^2$ is equivalent to the integration of the single-particle Wigner function with respect to position and momentum. The single-particle Wigner function is obtained by integration of the two-particle Wigner function $f_{w1,2}$ on one of the variables. Figure 5a shows the purity of the two wavepackets in the first scenario, where there is no Coulomb interaction. The first wavepacket is completely injected in the simulation region after about $60\, \mathrm fs$. Accordingly, the uncertainty rule is satisfied and the purity stabilizes at value 1, which prevails until the wavepacket starts to exit the simulation region, after about $190\,\mathrm fs$ from the beginning of the evolution. The injection of the second wavepacket begins $70 \,\mathrm fs$ after the first one, and is indicated by the blue curve. In the time interval between $t=140\,\mathrm fs$ and $t=190\,\mathrm fs$, highlighted by the dashed red lines, there is a window where both wavepackets evolve quantum mechanically, without interacting as two independent pure states. Figure 5b focuses on this time interval and shows the purity being perfectly equal to one. To give an insight into the numerical precision, it is important to note that the average value of the purity along the selected time interval is 0.99987 with a root mean square error (RMSE) of $9.8999 \cdot 10^{-5}$ for the first wavepacket, and an average value of 0.99891 with an RMSE of $2.9412 \cdot 10^{-4}$ for the second wavepacket.

**Fig. 6:**
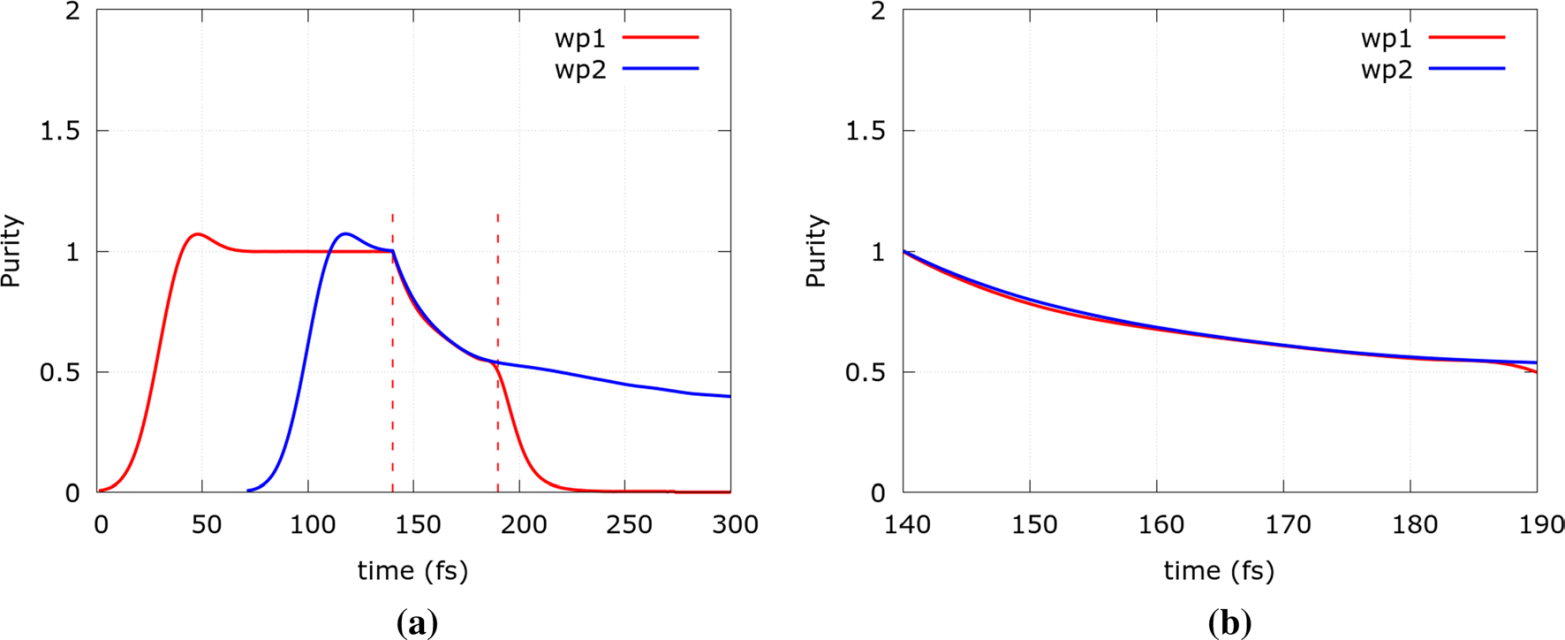
Time evolution of the purity of the two states in the Coulomb entangler for **a** the entire evolution time (dashed red lines indicate time interval shown in **b**), and **b** the time interval from $t=140\mathrm \,fs$ to from $t=190 \mathrm \, fs$ when both electrons are inside the quantum wire (Color figure online)

Figure 6 demonstrates the effect of the Coulomb repulsion. To better highlight the influence, the charge of each wavepacket is increased by an order of magnitude. Accordingly, at $t=140\, \mathrm fs$ the purity starts to drop at the beginning of the interaction. During the evolution the level of entanglement due to the Coulomb interaction increases, which is well-demonstrated by the continuous decline of the purity. As can be seen in Fig. 6b, the purity evolves equally for both electrons.

## Conclusions

We have investigated the effect of Coulomb interaction in the evolution of two electrons in a 2D quantum wire through the use of the computationally affordable Wigner formalism. The spectral decomposition of the input potential into a slowly varying classical component and a rapidly varying quantum mechanical component is analyzed in order to minimize the computational burden. It has been shown that the approximation of replacing the Wigner potential by an electrostatic force is reasonable, if the initial charge configuration is such that the electrons do not overlap in the beginning and do not cross over each other during the evolution. Purity is calculated to offer an insight into the Coulomb interaction between the electron–electron pairs. Comparing the purity of the injected pairs in two different scenarios verifies our approach to gauge quantum entanglement.

Coupling Wigner and Poisson equations paves the path for further research in different fields of quantum mechanics, including many-body investigations of particle evolutions. As future applications in the field of quantum transport will highly rely on efficient numerical approaches, the results obtained in this work will be particularly useful for future investigations of Coulomb interactions in the presence of quantum-entangled states.
